# Pregnancy-Associated Sacroiliitis following an Uncomplicated Pregnancy

**DOI:** 10.1155/2022/3596672

**Published:** 2022-05-04

**Authors:** Maryam Ahmadi, Farid Abiri, Somaieh Ahmadiani, Ensiyeh Jenabi

**Affiliations:** ^1^Clinical Research Development Unit of Fatemieh Hospital, Department of Gynecology, School of Medicine, Hamadan University of Medical Sciences, Hamadan, Iran; ^2^Orthopedic Surgury Resident, Iran University of Medical Sciences, Tehran, Iran; ^3^Clinical Research Development Unit of Fatemieh Hospital, Department of Gynecology, School of Medicine, Hamadan University of Medical Sciences, Hamadan, Iran; ^4^Autism Spectrum Disorders Research Center, Hamadan University of Medical Sciences, Hamadan, Iran

## Abstract

Pregnancy-associated sacroiliitis is infrequent during the peripartum period. Although pregnancy-associated pyogenic sacroiliitis rarely occurs, it is associated with significant morbidity and mortality. A timely diagnosis of the disease is challenging due to its nonspecific clinical symptoms. We reviewed a case that experienced an acute illness during pregnancy. The illness was manifested by localized pain in the hips or buttocks, sacroiliac joint tenderness, and debilitating hip pain during ambulation. Magnetic resonance imaging revealed little joint involvement, and the patient was treated with antibiotics, nonsteroidal anti-inflammatory drugs (NSAIDs), and glucocorticoids. The patient responded well to the therapy with marked improvement in her ambulation. Septic sacroiliitis should be considered in peripartum patients presenting with increased inflammatory markers and severe localized pain. Medical management is usually curative and without an adverse effect on pregnancy. Although we could not perform a biopsy to verify the cause of the disease, the patient's excellent response to the treatment confirmed our diagnosis.

## 1. Introduction

Hip and low back pain (LBP) frequently occur during pregnancy with a prevalence ranging from 30% to 78% [1]. Pain may originate from the lumbar region, hip, or sacroiliac joint (SIJ). LBP or buttocks usually has a nonspecific etiology and a self-limited course [2]. The risk factors for LBP include pelvic trauma, low age, multiparous, chronic LBP, and history of LBP in the previous pregnancy [3, 4].

However, certain conditions complicate the diagnosis, such as transient hip osteoporosis, hip avascular necrosis, sacral stress fracture, and sacroiliitis [2]. The sacroiliac joint disease usually presents with LBP that increases with ambulation [5]. Although most cases represent nonspecific arthritis, the sacroiliac joint can be seeded after bacteremia, resulting in a pyogenic course [2].

This complication is prevalent in injection drug users, although it may develop after any bacteremia. If the disease develops during pregnancy, it may pose a diagnostic challenge, as pain in the low back and buttocks is common and often nonspecific during pregnancy and the postpartum period [6, 7].

The incidence of pregnancy-associated sacroiliitis is low, with less than 20 cases reported in the literature occurring during pregnancy, during the puerperium, or after an abortion [2]. The pathophysiology of pregnancy-associated sacroiliitis may involve relaxation of pelvic ligaments during pregnancy, resulting in increases in pelvic movements and thus microtrauma to the joint surface [7]. Therefore, the physician may be susceptible to any transient bacteremia occurring in the context of pregnancy-induced immunosuppression [8].

Here, we reported a case of pregnancy-associated inflammatory sacroiliitis, which is rare and often underrecognized in daily clinical practice.

## 2. Case Presentation

A 24-year-old pregnant woman with a gestational age of about 35 weeks herself referred to an orthopedic emergency department. The patient suffered from sharp severe hip joint pain for a week. She was referred to a maternity hospital with a diagnosis of septic arthritis. After admission, the pain developed suddenly and radiated down to her right thigh. The pain was worsening over time. She regularly used local dairy products and did not take any specific medications. She had a successful vaginal delivery previously. A day after hospitalization, the labor pain began, and the vaginal delivery was conducted in the first hours of admission in the labor ward due to spontaneous labor pain. She suffered from pelvic pain and motion limitation of the hip, especially during the second stage of the delivery. The vaginal delivery had no dystocia regardless of the mother's pelvic pain. The newborn had a normal Apgar score (9/10). She was unable to ambulate postpartum due to excruciating hip pain. She had anorexia, chill and malaise, low-grade fever, and mild respiratory issues. She was hemodynamically stable in a normal range.

Urine and blood were collected and revealed an infective bladder, elevated C-reactive protein (CRP), negative rheumatoid factor (RF), negative 2-mercaptoethanol test (2 ME), negative blood culture (B/C), negative HIV, negative viral hepatitis, and elevated erythrocyte sedimentation rate (ESR) (135). A chest X-ray was performed, and it was normal. A hip magnetic resonance imaging (MRI) showed hyperlaxity of muscles around the right hip joint without evidence of bone damage. Mild collection of hematoma was noted, and the right sacroiliac joint showed fluid collection measuring 14 × 14 mm. The hips, femoral heads, and acetabulums were intact and showed normal congruency.

Lumbar magnetic resonance imaging (MRI) with and without contrast revealed normal lumbar spines. A pelvic magnetic resonance imaging (MRI) was performed and demonstrated an abnormal right sacroiliac joint signal sacroiliac joint (SIJ). SIG enhancement was revealed, which is in favor of arthritis. Empiric intravenous antibiotic therapy was administered to cover urinary tract infection (UTI), which was presumed to be the infection source. She was transferred to the rheumatology department for further treatment. The follow-up with her treating specialist revealed that she had improved significantly and regained her ability to function normally. She was discharged for a further follow-up of 10–20 days. An examination under anesthesia revealed no evidence of a perinatal abscess. On day 6 postpartum, blood cultures and a perinatal wound swab had grown group A *Streptococcus* (pyogenic); thus, her antibiotic regimen was rationalized to intravenous benzylpenicillin. However, she continued to have a fever and debilitating hip pain during ambulation.

**Figure 1:**
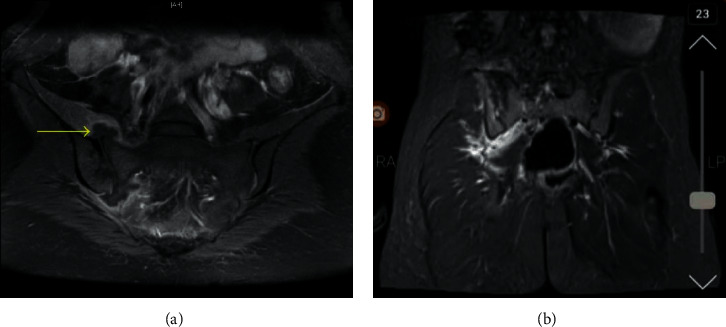
(a)-(b) Bilateral sacroiliitis with the right sacroiliac joint collection.

Furthermore, cultures of urine, blood, and perinatal swabs were negative, and a color Doppler ultrasound excluded pelvic deep venous thrombosis. On day 12 postpartum, she underwent a pelvic MRI scan, which revealed bilateral sacroiliitis with a right-sided sacroiliac joint collection (Figure 1). It was subsequently drained under computed tomography (CT) guidance, yielding 1 mL of purulent fluid and dramatically improving the patient's symptoms. She was discharged home on day 15 on a four-week intravenous benzylpenicillin course, followed by a six-week oral amoxicillin course.

After discharge, she was followed up and her gait and pain reduced day by day, and by the fourth week, her pain completely faded away.

An internal medicine physician observed her for two months after discharge from the hospital and prescribed her nonsteroidal anti-inflammatory drugs (NSAIDS) (ibuprofen 200 mg three times a day) and pregabalin (75 mg twice a day) for 4 weeks after discharging from the hospital. She showed full recovery and was found to ambulate freely with no sign of discomfort in her sacroiliac joint. The laboratory findings during admission and cure are given in Table 1.

## 3. Discussion

The sacroiliac joint is a nonweight-bearing joint with a small range of motion that has to endure extra burden during pregnancy due to biomechanical changes, leading to pubic instability, inflammation, bone edema, and stress fractures [9]. The latter can quickly occur if there is insufficient bone mass due to osteopenia or vitaminD deficiency [7, 10].

This case had severe pain, and her CRP was very high, leading us to include pyogenic sacroiliitis in the entry diagnosis. Pyogenic sacroiliitis may be accompanied by a history of intravenous drug abuse, infective endocarditis, urinary tract infection, abortion, or delivery [2, 11]. However, infections play an important role in this disease. The primary causative organisms are groups A and B *Streptococci* and *Staphylococcus* species [12]. Group A *Streptococcus* (GAS) is particularly important due to its mortality rate of 20–25%, a consequence of the toxic shock-like syndrome triggered by its exotoxin [13]. GAS can also cause rapid bone and joint destruction, resulting in chronic disability for survivors [14]. Although the timely diagnosis of pyogenic sacroiliitis is critical, it presents a diagnostic challenge. Moreover, one-third of patients with pyogenic sacroiliitis have a normal white cell count and are afebrile [2].

The most valuable investigations to establish this diagnosis are inflammatory markers, including almost universally elevated ESR and CRP levels, occasionally positive blood and urine cultures, and pelvic MRI. Bone edema, sclerosis, and erosions in SIJ are typical radiological changes similar to those in postpartum sacroiliitis [6]. Moreover, our case had a sign of upper tract infection. There were high ESR and CRP in her laboratory findings, which are in line with our diagnosis [6]. Empiric antibiotic regimens include vancomycin in combination with a third or fourth-generation cephalosporin (such as ceftriaxone, ceftazidime, or cefepime) and metronidazole. An alternative regimen consisting of vancomycin in combination with a 6 carbapenem is useful for coverage of staphylococci, Gram-negative bacilli, and anaerobes. Most patients remit completely in five months, but about 10% of them may experience more chronic persistent arthritis [6].

## 4. Conclusion

Hip and LBP are frequent during pregnancy or the postpartum period. A careful analysis of clinical, laboratory, and radiographic findings may help clinicians detect their underlying cause and tailor appropriate treatment.

## Figures and Tables

**Table 1:** The diagnosis test and inflammatory markers.

Time	ESR	CRP	PPD	RF	BC	CNP	Procalcitonin
At the beginning of the treatment	135	+	−	−	−	−	0.08
At the end of the treatment	35	+	−	−	−	−	?

ESR, erythrocyte sedimentation rate; CRP, C-reactive protein; PPD, purified protein derivative; RF, rheumatoid factor; BC, blood culture; CNP, C-type natriuretic peptide.

## Data Availability

The data used to support the findings of this study are available from the corresponding author upon request.
